# Phylogenetic structure of *Salmonella* Enteritidis provides context for a foodborne outbreak in Peru

**DOI:** 10.1038/s41598-020-78808-y

**Published:** 2020-12-16

**Authors:** Willi Quino, Junior Caro-Castro, Orson Mestanza, Carmen V. Hurtado, Maria L. Zamudio, Ronnie G. Gavilan

**Affiliations:** 1 Centro Nacional de Salud Pública, Instituto Nacional de Salud, Lima, Peru; 2 Escuela Profesional de Medicina Humana, Universidad Privada San Juan Bautista, Lima, Peru

**Keywords:** Microbiology, Molecular biology

## Abstract

*Salmonella* Enteritidis, an important foodborne zoonosis, has a dramatically increased number of cases around the world. To explore the phylogenetic structure of Peruvian *Salmonella* Enteritidis strains and their relationship with an outbreak occurred in 2018, we analyzed a comprehensive strains of *S.* Enteritidis received by the National Institute of Health during the period 2000–2018. A total of 180 strains were characterized by microbiological procedures, serotyping and whole genome sequencing. Based on genome sequences annotated, virulence factors and accessory genes were identified. Phylogenetic and population structure analysis were also analyzed based on SNPs. The phylogenetic analysis grouped the genomes into two well-supported clades that were consistent with population structure analysis. The clinical and food strains corresponding to the outbreak were included in the same cluster, which presented the *sdhA* gene, related to the increase of the virulence of this pathogen. The phylogenetic relationship of Peruvian *S.* Enteritidis suggests the presence of four *S.* enteritidis population with high epidemiological importance.

## Introduction

*Salmonella* spp. are one of the major causes of morbidity and mortality in children in many developing countries worldwide. The impact on global human health of non-typhoidal salmonellosis (NTS) is high, with an estimated of 93.8 million cases. 80.3 million of cases were related to food transmission^1^. In Latin America, Asia and Africa, the reported incidence of *Salmonella* spp. is 200 to 500 cases per 100,000 inhabitants annually. Since the transmission of *Salmonella* spp. from person to person is uncommon, food is considered the main source of human exposure, with an estimated 95% of infections related to animal source food^2^.

*S. enterica* subsp. *enterica* serovar Enteritidis (*S.* Enteritidis) is a serovar that colonizes the reproductive tract of chickens, including other birds and wild animal, usually asymptomatically^3^.In addition, the bacteria use bird eggs as the main vehicle of infection, which can be contaminated internally and on the outer shell surface. Internal contamination is usually due to the result of bacterial penetration through the egg shell via microscopic cracks. On the other hand, external contamination occurs before oviposition, caused by infection of the bird's reproductive organs^4^.In addition, there are other factors that increase *S.* Enteritidis infection among humans, such as cross contamination of raw and prepared foods^5^, as well as the existence of asymptomatic infected food handlers^6^. For all these reasons, an emerging foodborne zoonosis caused by *S.* Enteritidis has increased dramatically since the 1980s, when *S.* Enteritidis replaced *S.* Typhimurium as the primary cause of salmonellosis worldwide^7^. In fact, recent studies in Latin America from 2005 to 2009 reported a higher *S.* Enteritidis isolation rate (51%) compared to 21% *S.* Typhimurium^8^.

Whole genome sequencing (WGS) is currently being widely used for the surveillance of foodborne pathogens. Prospectively, the typing of *S.* Enteritidis strains could identify possible outbreaks in real time. Phylogenetic methods that explore the relationship between microbial genomes have been used for the study of emergence, geographic spread and transmission of infections^9^, as shown by various investigations related to this pathogen^3,10,11^. Therefore, in this study we explored the phylogenetic structure of Peruvian *S.* Enteritidis strains from the last 20 years and its relationship with an outbreak occurred in Callao, Peru, in 2018.

## Results

### Identification of *Salmonella* enteritidis

**Figure 1:**
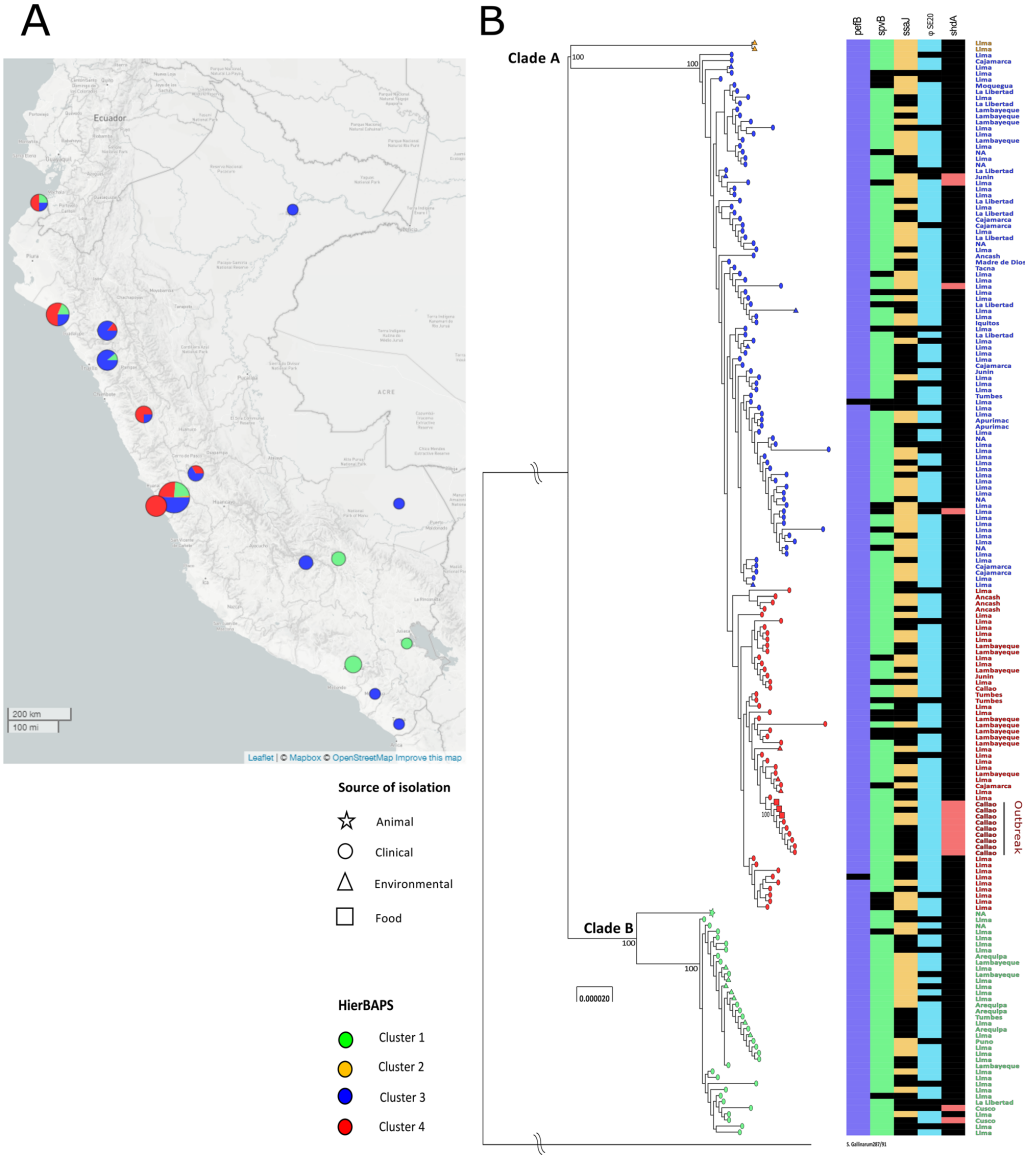
Phylogenomic analysis of *Salmonella* Enteritidis isolated in Peru from different sources. (**A**) Map of Peru and geographical locations of *S.* Enteritidis isolation generated using Microreact tool. The colors in each circular graph represent the four populations obtained with hierBAPS. (**B**) Maximum likelihood tree constructed based on LCBs, using the GTR + G model. The supporting nodes were calculated with a total of 1000 bootstrap. The shapes indicate the source of isolation and the colors are based on HierBAPS populations (see caption). In addition, the geographical origin of the strains and the presence or absence of the virulence factors and accessory genome are indicated next to each strain (Colored: Presence of the gene, Black: Absence of the gene). Both figures were obtained with the Microreact tool.

In the study, all 180 strains, including strains obtained from the outbreak, were identified as *S.* Enteritidis by conventional microbiological procedures, whereby all the strains were defined by the formula 1,9,12: g, m:—(Supplementary material 1). The geographical area where each strain was isolated can be seen in Fig. 1A, colored by the populations obtained with hierBAPS.

### Analysis of *Salmonella* Enteritidis genomes

Genome sequencing analysis was performed with all 180 strains, obtaining an average of 3.3 million reads, 77 contigs and a G + C percentage of 52.0%. The average size of the genomes was 4.8 Mb. MLST analysis determined that all sequenced strains of *S*. Enteritidis corresponded to the sequence type 11 (ST11), which is the most common ST of *S.* Enteritidis (http://enterobase.warwick.ac.uk/species/senterica/search_strains).

The alignment of the 180 genome sequences gave us a core genome of 65.7%, which presented 6208 SNPs within locally colinear blocks of more than 3.2 Mb. The ratio of recombination and mutation was R/θ = 0.07, whereas the ratio of effects of recombination and mutation was r/m = 0.26, having low recombination events in these bacteria.

The Maximum Likelihood tree (Fig. 1B) revealed two well-defined clades: clade A, encompassing the majority of strains (143), including those belonging to the outbreak, and clade B, with 37 strains. The phylogeny was divided into four populations using hierBAPS, all with 100% of bootstrap. Clade A is composed only by population 1 (green), whereas clade B comprises population 2 (orange), 3 (blue) and 4 (red).

Population 1 (green) contains strains from clinical, environmental and animal sources from 2001 to 2013. It is noteworthy that only strains isolated from 2001 to 2002 belong to population 1, corresponding to 48.6%. Population 2 (orange) comprises two environmental strains isolated in 2017, whereas population 3 (blue) contains clinical (94.3% of the total number of strains) and environmental strains, obtained from 2000 to 2017. Population 4 (red), located in the branch most internal one, comprises clinical, food and environmental strains obtained from 2008 to 2018. The recent outbreak strains are located inside population 4.

### Analysis of virulence factors and accessory genome

Few genes gave relevant information that allowed us to find differences between clades. *pefB* and *spvB* genes were found in almost all Peruvian strains (98.9% and 84.4%, respectively), and did not show any evident pattern. A similar situation was found with *ssaJ* gene, which was detected in half of strains evaluated (50.6%). However, the *shdA* gene was absent in the majority of studied strains (91.7%), with the particular exception of those belonging to the 2018 outbreak, which presented it within its genome. Regarding to phage regions φSE20, they were found in a big number of Peruvian strains (80.6%). The presence or absence of these genes can be visualized attached to each strain in Fig. 1B.

## Discussion

*Salmonella* Enteritidis is one of the main causes of food-related illness worldwide, facilitated by its ability to colonize and infect a wide range of hosts, especially through eggs and products made with them^22^. In Peru, there are few studies and records related to the study of the epidemiology of this serovar, and little information related to phylogenetic and molecular studies^23,24^. Therefore, this study offers a first look at the phylogenetic structure of Peruvian *S.* Enteritidis strains received by INS from different regions of Peru as part of national surveillance.

In this work, genomes of *S*. Enteritidis isolated in Peru from diverse geographical origins and diverse sources of isolation in a period of almost 20 years were analyzed, which presented a very similar genetic profile, independently of the source from which they were obtained or their host. It is known that *S.* Enteritidis is a highly clonal serovar within the genus *Salmonella*^25^. In our study, all strains belonged to the same ST and showed low rate of recombination, which reflects the high degree of clonality of this circulating serovar through Peru. The maximum likelihood analysis of Peruvian strains shows two clearly differentiated clades, although no specific grouping depends on the source of isolation: clade A, whose branch is close to the outgroup *S.* Gallinarum, and clade B, whose branch is far from the reference used as outgroup. This result is a pattern that is often repeated in many phylogenetic works related to this serovar^11,26^; consequently, it could be an evidence that two lineages of *S.* Enteritidis are currently circulating in Peru.

A relevant fact is that strains from different sources can be phylogenetically related. This is especially relevant in the analysis of the most internal population obtained with the hierBAPS (population 4, red color). The isolated strains from mayonnaise are phylogenetically related to the strains obtained from the patients of the outbreak, which incriminates mayonnaise as a transport vehicle of the disease. In addition, inside the genetic information of strains from the outbreak, we emphasize the presence of *shdA* gene. This gene encodes an intestinal colonization adhesin, homologous to various adhesins found in other pathogens such as diffuse adhesion *E. coli* (DAEC), *S.* Typhimurium and *Shigella flexneri*. Also, it is commonly associated with *Salmonella* serovars isolated from poultry, being involved in the expansion of *S.* enterica host range within warm-blooded vertebrates^27^. Besides, it is the only known determinant for persistence bacterial in the cecum, allowing an efficient and prolonged excretion of it in the feces, which contributes to a greater virulence of this pathogen^28,29^.

A detail in the outermost population (population 1, green color) is remarked: the presence of isolated strains from 2001–2002, which make up half of strains from this group. These strains are not found in the other populations. In addition, other members of this population after the indicated years suggest that the circulation of strains of this lineage is still occurring in a small number. This could explain the difficulty to trace them the year by year. Inside population 2 (orange color), there are only two strains of environmental source. This could indicate a high rate of variation outside of an animal host.

Another interesting result was the presence of the phage φSE20 regions, which was found in 80.4% of the strains, and located in the four populations obtained in the analysis. Shah et al*.*^30^ define it as a prophage that transports genes involved in the pathogenicity of *Salmonella*, which is suspected to be related to the emergence of *S.* Enteritidis worldwide. Feasey et al*.*^31^ found the presence of these regions within what they called the “global epidemic clade”. Subsequently, in a study made in Brazil, the presence of these regions was reported in all strains after 1994, year in which the increase of serovar Enteritidis strains began within this country, concluding that the emergence of the infection by this serovar could be related to that phage^3^. Due to previous reports, and the findings of the present study, the emergence of *S.* Enteritidis in Peru could be related to the presence of strains that include phage regions φSE20 within its genome. To have more support for this hypothesis, it is required to obtain samples prior to 1995, and to verify the majority absence of these regions in those strains.

In a complementary way, it is observed that the distribution of the strains by geographical region of Peru does not follow a pattern, because strains from different populations are distributed indistinctly between the different regions. Therefore, it can be deduced that both lineages would be causing infection throughout the territory. This same distribution that does not follow any clear distribution was also previously observed in Brazil and Chile^3,11^.

The study presents the limitation of not having isolated strains prior to the year 2000 and from the 25 regions of Peru, which would have helped significantly to represent the overall picture of the country. Also, it could help to reinforce the hypothesis of Campioni et al*.*^3^, where the majority absence of genes belonging to phage φSE20 was highlighted, a stage called pre-pandemic by these authors.

In conclusion, the phylogenetic relationship of Peruvian strains from 2000 to 2018 suggests the presence of two well-defined clades and, therefore, two different lineages of *S*. Enteritidis causing disease in humans. The presence of four well-defined populations with particular characteristics suggest discrete groups of clinical strains that are being transmitted in the different geographical areas without showing any clear pattern of geographical distribution is remarked. Finally, the outbreak occurred in 2018 in Callao was due to a highly virulent strain, evidenced by the genomic presence of the *shdA* gene, whose frequency rate in strains from the period of time evaluated is low.

## Methods

### Sampling and population

In this study, we included a total of 180 strains received by the National Reference Laboratory of Enteropathogens of the National Health Institute (in Spanish, Instituto Nacional de Salud, INS) from different parts of Peru during the years 2000–2018, which were previously characterized as *Salmonella* spp.

Nine strains were isolated in order to study a possible outbreak occurred in Callao, Peru. These nine strains included five isolated from patients with signs and symptoms of acute diarrheal disease after consuming at a restaurant in Callao, Peru, occurred in 2018, and three strains from the possible source of contamination, mayonnaise prepared in the same place.

### Ethical statement

The use of human biological material was approved by the Institutional Research Ethics Committee/INS with register No 20516–2018. The patient information was completely anonymized in this study.

### Culturing and biochemical confirmation

All 180 strains were recovered using a selective media. First, all the strains were inoculated in Trypticase Soy Broth (Merck, Germany) at 37 °C from six to eight hours. Subsequently, they were grown on SS Agar plates (Salmonella Shigella Agar; Merck, Germany) and incubated at 37 °C from 18 to 24 h. The genus *Salmonella* was confirmed using conventional biochemical tests. These strains were analyzed with O and H agglutination antisera (Bio Merieux, France) following the Kauffmann-White scheme for the identification of serovars^12^.

### Preparation of libraries and sequencing

All 180 strains identified by serotyping as *Salmonella* Enteritidis were selected for sequencing. The DNA extraction of those strains was carried out using the DNeasy Blood & Tissue kit (Qiagen, Germany) following the manufacturer's instructions. The genomic DNA samples previously obtained were quantified by fluorometry (Quibit 3.0 Invitrogen, Malaysia). The sequencing libraries was performed using the Nextera XT Library Preparation Kit (Illumina, USA), and genomic sequencing using MiSeq (Illumina, USA).

### Analysis of genomic data

The quality of each obtained sequence was evaluated through the software FastQC v0.11.5 (https://www.bioinformatics.babraham.ac.uk/projects/fastqc). Adapters and low-quality bases were removed using the program Trimmomatic v0.38^13^. The sequences were assembled de novo using the pipeline A5-miseq v20160825^14^. The identification of genus and detection of possible contaminated contigs were made with the program Kraken v1.0^15^ using Minikraken database. Inclusion criteria include genomes with had less than 300 contigs and 92% of them belonging to *Salmonella* spp. The genotype of each sequence was identified using the software Multilocus Sequence Typing (MLST) v2.10.

For the phylogenetic study, the genome of *S.* Gallinarum strain 287/91 (accession number AM933173.1) stored in the GenBank database (http://www.ncbi.nlm.nih.gov) was used as outgroup. Subsequently, the genome alignment was performed with the program Parsnp v1.2, whose result, locally collinear blocks (LCB) containing the SNPs, were submitted to the program HarvestTools v1.2^16^ to extract the alignment.

The phylogeny was performed by the Maximum Likelihood method using the software RaxML v8.0^17^, built using the GTR + G model, with supporting nodes calculated using 1000 of bootstrap. The removal of recombinant regions was done using the program ClonalFrameML v1.11^18^. The analysis of the population structure was evaluated using hierBAPS algorithm implemented in R language v3.2.3 package^19^, using following parameters: a) include singleton SNPs, b) maximum depth = 2 and c) number of populations = 20. Finally, the visualization of results was performed using the online tool Microreact and accessible following https://microreact.org/project/9G7UFQLRAwiS4S1J13M69C^20^.

### Analysis of virulence factors and accessory genome

The BLAST tool was used to search virulence factors using VFDB (Available at http://www.mgc.ac.cn/VFs/) such as *pefB* (fimbrial regulatory plasmid gene), *shdA* (intestinal colonization adhesin gene), *spvB* (intracellular ADP-ribosylase toxin gene) and *ssaJ* (gene of a major component of Salmonella pathogenicity island 2 type III secretion system), in addition to regions of *S.* Enteritidis φST64B-like prophage φSE20.

The prediction of coding sequences for each library was performed using the program Prodigal v2.6.3^21^. The homologous genes of the sequences were identified from the genes of the reference genome, with a > 90% identity using BLAST algorithm and > 60% of alignment coverage to the reference. The code used for the annotation is available at http://github.com/OrsonMM/Blast-score-ratio-for-genomics. Genomic data have been deposited in GenBank (BioProject ID: PRJNA552561).

## Supplementary information

Supplementary Information 1.
