# Supplementary material for: Phylogenetic structure of Salmonella Enteritidis provides context for a foodborne outbreak in Peru
Source: Sci Rep. 2020 Dec 16;10:22080. doi: 10.1038/s41598-020-78808-y (PMC7745040; doi:10.1038/s41598-020-78808-y)
Supplement: Supplementary file 1 — Supplementary Information 1. [file 41598_2020_78808_MOESM1_ESM.pdf]

Phylogenetic structure of *Salmonella* Enteritidis provides context for a foodborne outbreak in Peru

Willi Quino[1], Junior Caro Castro[1], Orson Mestanza[1], Carmen Veronica Hurtado[1], Maria Luz Zamudio[1], Ronnie G. Gavilan[1*]

[1]Instituto Nacional de Salud, Lima, Peru. ***email:** rgavilan@ins.gob.pe

Supplementary information file 1. Metadata for the 180 *Salmonella* Enteritidis strains isolated in Peru in this study.

ID	INS code	NCBI Accesion number	Year of isolation	Source of isolation	Peruvian region of isolation	HierBAPS análisis population	φSE20 regions	*spvB* genes	*shdA* genes	*ssaJ* genes	*pefB* genes
FD01848527	2.558-2001	VNEW00000000	2001	Clinical	Puno	1	-	+	-	+	+
FD01848531	2.615-2001	VMYN00000000	2001	Clinical	Arequipa	1	+	+	-	+	+
FD01848532	2.629-2001	VMYL00000000	2001	Clinical	Arequipa	1	+	+	-	-	+
FD01848534	1.188-2002	VNED00000000	2002	Clinical	Lima	1	+	+	-	+	+
FD01848537	2.089-2002	VNFA00000000	2002	Clinical	Tumbes	1	+	+	-	-	+
FD01848539	1.084-2002	VNCN00000000	2002	Clinical	Lima	1	+	+	-	-	+
FD01848540	3.081-2002	VNBG00000000	2002	Environmental	Lima	1	-	+	-	+	+
FD01848542	2.137-2002	VMYO00000000	2002	Clinical	Arequipa	1	+	+	-	+	+
FD01848543	2.139-2002	VMYM00000000	2002	Clinical	Arequipa	1	+	+	-	-	+
FD01848544	2.149-2002	VNAK00000000	2002	Clinical	Lambayeque	1	+	+	-	+	+
FD01848545	3.153-2002	VNBH00000000	2002	Environmental	Lima	1	-	+	-	+	+
FD01848546	3.154-2002	VNEE00000000	2002	Environmental	Lima	1	+	+	-	+	+
FD01848547	3.155-2002	VNEF00000000	2002	Environmental	Lima	1	+	+	-	+	+
FD01848548	2.225-2002	VNAE00000000	2002	Clinical	Lambayeque	1	+	+	-	-	+
FD01848549	2.227-2002	VMZW00000000	2002	Clinical	Lambayeque	1	-	+	-	+	+
FD01848550	3.250-2002	VNEG00000000	2002	Environmental	Lima	1	+	+	-	+	+
FD01848551	3.251-2002	VNCO00000000	2002	Environmental	Lima	1	+	+	-	-	+
FD01848553	1.363-2002	VNEH00000000	2002	Clinical	Lima	1	+	+	-	+	+
FD01848619	2.840-1999	VMZN00000000	1999	Clinical	La Libertad	1	-	+	-	-	+
FD01848703	2.842-2001	VMZH00000000	2001	Clinical	Cusco	1	+	+	+	-	+
FD01848708	2.850-2001	VMZG00000000	2001	Clinical	Cusco	1	-	+	+	-	+
FD01848711	1.960-2001	VNCP00000000	2001	Clinical	Lima	1	+	-	-	-	+
FD01851395	1.159-2010	VNEI00000000	2010	Clinical	Lima	1	+	+	-	+	+
FD01851396	1.167-2010	VNCQ00000000	2010	Clinical	Lima	1	+	+	-	-	+
FD01851402	3.195-2010	VNCR00000000	2010	Environmental	Lima	1	+	+	-	-	+
FD01852575	1.463-2013	VNAV00000000	2013	Clinical	Lima	1	-	+	-	-	+
FD01852680	1.674-2012	VNEJ00000000	2012	Clinical	Lima	1	+	+	-	+	+
FD01852682	1.688-2012	VNCS00000000	2012	Clinical	Lima	1	+	+	-	-	+
FD01852714	1.123-2013	VNBI00000000	2013	Clinical	Lima	1	-	-	-	+	+

FD01852716	1.125-2013	VNAW00000000	2013	Clinical	Lima	1	-	+	-	-	+
FD01852722	1.134-2013	VNCT00000000	2013	Clinical	Lima	1	+	+	-	-	+
FD01852725	1.142-2013	VNCU00000000	2013	Clinical	Lima	1	+	+	-	-	+
FD01852726	1.143-2013	VNAX00000000	2013	Clinical	Lima	1	-	+	-	-	+
FD01852859	1.324-2010	VNAY00000000	2010	Clinical	Lima	1	-	+	-	-	+
FD01852894	1.546-2010	VNEK00000000	2010	Clinical	Lima	1	+	+	-	+	+
FD01852909	1.569-2010	VNEV00000000	2010	Clinical	NA	1	+	+	-	+	+
SRR4098714	NA	SRR4098714	2008	Animal	NA	1	+	+	-	-	+
FD01875488	3.569-2017	VNEL00000000	2017	Environmental	Lima	2	+	+	-	+	+
FD01875498	3.572-2017	VNEM00000000	2017	Environmental	Lima	2	+	+	-	+	+
FD01846462	1.127-2017	VNCV00000000	2017	Clinical	Lima	3	+	+	-	+	+
FD01848592	2.169-2005	VNEX00000000	2005	Clinical	Tacna	3	+	+	-	-	+
FD01848595	1.190-2005	VNCW00000000	2005	Clinical	Lima	3	+	+	-	+	+
FD01848618	2.848-1999	VMZO00000000	1999	Clinical	La Libertad	3	+	+	-	-	+
FD01848626	2.352-1999	VMZM00000000	1999	Clinical	La Libertad	3	-	+	-	-	+
FD01848665	4.064-2000	VMZJ00000000	2000	Environmental	Junin	3	-	+	+	+	+
FD01848668	2.390-2000	VNCX00000000	2000	Clinical	Lima	3	+	+	-	+	+
FD01848669	2.391-2000	VNCY00000000	2000	Clinical	Lima	3	+	+	-	+	+
FD01848671	1.235-2000	VNBJ00000000	2000	Clinical	Lima	3	+	-	+	+	+
FD01848685	2.004-2001	VMZP00000000	2001	Clinical	La Libertad	3	+	+	-	-	+
FD01848686	2.015-2001	VMZT00000000	2001	Clinical	La Libertad	3	+	+	-	+	+
FD01848688	2.288-2001	VNEO00000000	2001	Clinical	Moquegua	3	+	-	-	+	+
FD01851321	3.052-2009	VNCZ00000000	2009	Environmental	Lima	3	+	+	-	+	+
FD01851329	2.163-2009	VMYQ00000000	2009	Clinical	Cajamarca	3	-	+	-	+	+
FD01851339	1.322-2009	VNAM00000000	2009	Clinical	Lima	3	-	-	-	-	+
FD01851347	3.454-2009	VNBL00000000	2009	Environmental	Lima	3	+	+	-	-	+
FD01851367	3.084-2008	VNBM00000000	2008	Environmental	Lima	3	+	+	-	-	+
FD01851370	2.171-2008	VNEN00000000	2008	Clinical	Madre de Dios	3	+	+	-	-	+
FD01851372	2.226-2008	VMYP00000000	2008	Clinical	Cajamarca	3	-	+	-	-	+
FD01851382	1.719-2009	VNAN00000000	2009	Clinical	Lima	3	-	+	-	-	+
FD01851384	1.780-2009	VNBN00000000	2009	Clinical	Lima	3	+	+	-	-	+
FD01851393	1.152-2010	VNBO00000000	2010	Clinical	Lima	3	+	+	-	-	+
FD01851421	2.832-2007	VNAF00000000	2007	Clinical	Lambayeque	3	+	+	-	+	+
FD01851423	1.864-2007	VNDA00000000	2007	Clinical	Lima	3	+	+	-	+	+
FD01851435	2.962-2007	VMZK00000000	2007	Clinical	Junin	3	+	+	-	-	+
FD01851437	1.1148-2007	VNDB00000000	2007	Clinical	Lima	3	+	+	-	+	+
FD01851438	1.1194-2007	VNBP00000000	2007	Clinical	Lima	3	+	+	-	-	+
FD01851504	1.599-2005	VNDC00000000	2005	Clinical	Lima	3	+	+	-	+	+
FD01851523	2.011-2006	VNAG00000000	2006	Clinical	Lambayeque	3	+	+	-	+	+
FD01851524	2.012-2006	VMZX00000000	2006	Clinical	Lambayeque	3	+	+	-	-	+
FD01851525	2.015-2006	VNAH00000000	2006	Clinical	Lambayeque	3	+	+	-	+	+
FD01851540	2.301-2006	VMZQ00000000	2006	Clinical	La Libertad	3	+	+	-	-	+

FD01851551	2.469-2006	VMZI00000000	2006	Clinical	Iquitos	3	+	+	-	+	+
FD01851556	3.519-2006	VNBQ00000000	2006	Environmental	Lima	3	+	+	-	-	+
FD01851565	1.064-2007	VNDD00000000	2007	Clinical	Lima	3	+	+	-	+	+
FD01851569	2.125-2007	VMZR00000000	2007	Clinical	La Libertad	3	+	-	-	-	+
FD01851572	1.193-2007	VNBK00000000	2007	Clinical	Lima	3	+	+	+	+	+
FD01851573	1.194-2007	VNBR00000000	2007	Clinical	Lima	3	+	-	-	-	+
FD01851574	1.195-2007	VNAZ00000000	2007	Clinical	Lima	3	-	-	-	+	+
FD01851581	1.281-2007	VNDE00000000	2007	Clinical	Lima	3	+	-	-	+	+
FD01851591	1.490-2007	VNDF00000000	2007	Clinical	Lima	3	+	+	-	+	+
FD01852463	1.167-2015	VNBA00000000	2015	Clinical	Lima	3	-	+	-	+	+
FD01852486	1.458-2015	VNDG00000000	2015	Clinical	Lima	3	+	+	-	+	+
FD01852507	2.205-2016	VMYS00000000	2016	Clinical	Cajamarca	3	+	+	-	+	+
FD01852516	1.303-2016	VNBB00000000	2016	Clinical	Lima	3	-	-	-	+	+
FD01852527	1.428-2016	VNDH00000000	2016	Clinical	Lima	3	+	-	-	+	+
FD01852540	1.010-2017	VNEP00000000	2017	Clinical	NA	3	+	+	-	-	+
FD01852547	1.024-2017	VNAL00000000	2017	Clinical	Lima	3	-	-	+	+	+
FD01852560	1.376-2013	VNDI00000000	2013	Clinical	Lima	3	+	+	-	+	+
FD01852561	1.378-2013	VNES00000000	2013	Clinical	NA	3	+	-	-	+	+
FD01852563	2.390-2013	VMYK00000000	2013	Clinical	Apurimac	3	+	+	-	+	+
FD01852565	2.393-2013	VMYJ00000000	2013	Clinical	Apurimac	3	-	+	-	-	+
FD01852577	1.515-2013	VNDJ00000000	2013	Clinical	Lima	3	+	+	-	+	+
FD01852580	1.537-2013	VNET00000000	2013	Clinical	NA	3	+	+	-	+	+
FD01852581	1.577-2013	VNEQ00000000	2013	Clinical	NA	3	+	+	-	-	+
FD01852596	2.196-2014	VMYT00000000	2014	Clinical	Cajamarca	3	+	+	-	+	+
FD01852609	2.578-2014	VMYU00000000	2014	Clinical	Cajamarca	3	+	+	-	+	+
FD01852618	1.670-2014	VNEU00000000	2014	Clinical	NA	3	+	-	-	+	+
FD01852623	1.693-2014	VNDK00000000	2014	Clinical	Lima	3	+	+	-	+	+
FD01852628	1.002-2015	VNBC00000000	2015	Clinical	Lima	3	-	+	-	+	+
FD01852632	1.029-2015	VNDL00000000	2015	Clinical	Lima	3	+	+	-	+	+
FD01852635	1.038-2015	VNBS00000000	2015	Clinical	Lima	3	+	+	-	-	+
FD01852676	1.614-2012	VNDM00000000	2012	Clinical	Lima	3	+	+	-	+	+
FD01852677	1.615-2012	VNAO00000000	2012	Clinical	Lima	3	-	+	-	-	+
FD01852717	1.127-2013	VNBD00000000	2013	Clinical	Lima	3	-	+	-	+	+
FD01852720	1.130-2013	VNBE00000000	2013	Clinical	Lima	3	-	+	-	+	+
FD01852727	1.145-2013	VNBT00000000	2013	Clinical	Lima	3	+	+	-	-	+
FD01852728	1.147-2013	VNAP00000000	2013	Clinical	Lima	3	-	-	-	-	+
FD01852803	1.002-2012	VNAQ00000000	2012	Clinical	Lima	3	-	-	-	-	+
FD01852804	2.012-2012	VMYG00000000	2012	Clinical	Ancash	3	+	+	-	+	+
FD01852810	1.063-2012	VNBU00000000	2012	Clinical	Lima	3	+	-	-	-	-
FD01852811	1.064-2012	VNAR00000000	2012	Clinical	Lima	3	-	+	-	-	+
FD01852815	2.171-2012	VNEZ00000000	2012	Clinical	Tumbes	3	+	+	-	-	+
FD01852820	1.211-2012	VNER00000000	2012	Clinical	NA	3	+	+	-	-	+
FD01852829	2.331-2012	VMYR00000000	2012	Clinical	Cajamarca	3	+	+	-	-	+

FD01852830	1.335-2012	VNDN00000000	2012	Clinical	Lima	3	+	+	-	+	+
FD01852831	1.337-2012	VNDO00000000	2012	Clinical	Lima	3	+	+	-	+	+
FD01852834	1.344-2012	VNBV00000000	2012	Clinical	Lima	3	+	+	-	-	+
FD01852836	1.350-2012	VNBW00000000	2012	Clinical	Lima	3	+	+	-	-	+
FD01852853	1.253-2010	VNBX00000000	2010	Clinical	Lima	3	+	+	-	-	+
FD01852854	1.259-2010	VNBY00000000	2010	Clinical	Lima	3	+	+	-	-	+
FD01852855	1.265-2010	VNBZ00000000	2010	Clinical	Lima	3	+	+	-	-	+
FD01852920	1.595-2010	VNCA00000000	2010	Clinical	Lima	3	+	+	-	-	+
FD01852926	1.631-2010	VNDP00000000	2010	Clinical	Lima	3	+	+	-	+	+
FD01852927	2.646-2010	VMZS00000000	2010	Clinical	La Libertad	3	+	+	-	-	+
FD01852928	2.649-2010	VMZU00000000	2010	Clinical	La Libertad	3	+	+	-	+	+
FD01852934	1.001-2011	VNDQ00000000	2011	Clinical	Lima	3	+	+	-	+	+
FD01872662	1.184-2017	VNDR00000000	2017	Clinical	Lima	3	+	+	-	+	+
892	6.892-2018	VMYW00000000	2018	Clinical	Callao	4	+	+	+	-	+
893	6.893-2018	VMZC00000000	2018	Clinical	Callao	4	+	+	+	+	+
894	6.894-2018	VMYX00000000	2018	Clinical	Callao	4	+	+	+	-	+
895	6.895-2018	VMYY00000000	2018	Clinical	Callao	4	+	+	+	-	+
898	6.898-2018	VMZA00000000	2018	Clinical	Callao	4	+	+	+	-	+
902	6.902-2018	VMYZ00000000	2018	Clinical	Callao	4	+	+	+	-	+
4001	4.001-2019	VMZD00000000	2018	Food	Callao	4	+	+	+	+	+
4002	4.002-2019	VMZE00000000	2018	Food	Callao	4	+	+	+	+	+
4003	4.003-2019	VMZB00000000	2018	Food	Callao	4	+	+	+	-	+
FD01851371	1.185-2008	VNCB00000000	2008	Clinical	Lima	4	+	-	-	-	+
FD01851449	3.124-2008	VNDS00000000	2008	Clinical	Lima	4	+	-	-	+	+
FD01851464	1.436-2008	VNDT00000000	2008	Clinical	Lima	4	+	+	-	+	+
FD01851475	2.620-2008	VMZL00000000	2008	Clinical	Junin	4	+	+	-	+	+
FD01851486	2.742-2008	VMZY00000000	2008	Clinical	Lambayeque	4	+	+	-	-	+
FD01851531	2.166-2006	VNAI00000000	2006	Clinical	Lambayeque	4	+	+	-	+	+
FD01852472	1.333-2015	VNDU00000000	2015	Clinical	Lima	4	+	+	-	+	+
FD01852477	6.383-2015	VNCC00000000	2015	Clinical	Lima	4	+	+	-	-	+
FD01852480	2.440-2015	VMZZ00000000	2015	Clinical	Lambayeque	4	+	+	-	-	+
FD01852481	2.442-2015	VNAA00000000	2015	Clinical	Lambayeque	4	+	+	-	-	+
FD01852482	1.453-2015	VNCD00000000	2015	Clinical	Lima	4	+	+	-	-	+
FD01852487	1.472-2015	VNDV00000000	2015	Clinical	Lima	4	+	+	-	+	+
FD01852493	1.533-2015	VNDW00000000	2015	Environmental	Lima	4	+	+	-	+	+
FD01852526	2.400-2016	VMYV00000000	2016	Clinical	Cajamarca	4	+	-	-	+	+
FD01852550	3.054-2017	VNCE00000000	2017	Environmental	Lima	4	+	+	-	-	+
FD01852551	3.055-2017	VNCF00000000	2017	Environmental	Lima	4	+	+	-	-	+
FD01852555	2.358-2013	VMYF00000000	2013	Clinical	Ancash	4	+	+	-	-	+
FD01852584	1.603-2013	VNDX00000000	2013	Clinical	Lima	4	+	+	-	+	+
FD01852585	1.604-2013	VNCG00000000	2013	Clinical	Lima	4	+	+	-	-	-
FD01852588	1.036-2014	VNDY00000000	2014	Clinical	Lima	4	+	+	-	+	+
FD01852606	1.489-2014	VNCH00000000	2014	Clinical	Lima	4	+	+	-	-	+

FD01852607	1.490-2014	VNDZ00000000	2014	Clinical	Lima	4	+	+	-	+	+
FD01852629	1.009-2015	VNEA00000000	2015	Clinical	Lima	4	+	-	-	+	+
FD01852636	1.060-2015	VNBF00000000	2015	Clinical	Lima	4	-	-	-	+	+
FD01852643	1.086-2015	VNCI00000000	2015	Clinical	Lima	4	+	+	-	-	+
FD01852648	1.122-2015	VNEB00000000	2015	Clinical	Lima	4	+	-	-	+	+
FD01852652	1.388-2012	VNCJ00000000	2012	Clinical	Lima	4	+	+	-	-	+
FD01852663	1.576-2012	VNCK00000000	2012	Clinical	Lima	4	+	-	-	-	+
FD01852667	2.582-2012	VNAB00000000	2012	Clinical	Lambayeque	4	+	-	-	-	+
FD01852668	2.583-2012	VNAC00000000	2012	Clinical	Lambayeque	4	+	-	-	-	+
FD01852669	2.584-2012	VNAJ00000000	2012	Clinical	Lambayeque	4	+	+	-	+	+
FD01852670	2.587-2012	VMZV00000000	2012	Clinical	Lambayeque	4	-	-	-	-	+
FD01852672	2.590-2012	VNAD00000000	2012	Clinical	Lambayeque	4	+	+	-	-	+
FD01852696	1.790-2012	VNAS00000000	2012	Clinical	Lima	4	-	+	-	-	+
FD01852698	1.798-2012	VNCL00000000	2012	Clinical	Lima	4	+	+	-	-	+
FD01852708	1.114-2013	VNAT00000000	2013	Clinical	Lima	4	-	+	-	-	+
FD01852711	1.118-2013	VNEC00000000	2013	Clinical	Lima	4	+	+	-	+	+
FD01852745	2.355-2013	VMYH00000000	2013	Clinical	Ancash	4	+	+	-	+	+
FD01852746	2.357-2013	VMYI00000000	2013	Clinical	Ancash	4	+	+	-	+	+
FD01852817	2.179-2012	VNEY00000000	2012	Clinical	Tumbes	4	-	-	-	-	+
FD01852819	2.181-2012	VNFB00000000	2012	Clinical	Tumbes	4	+	+	-	+	+
FD01852827	1.298-2012	VMZF00000000	2012	Clinical	Callao	4	+	+	-	+	+
FD01852828	1.327-2012	VNCM00000000	2012	Clinical	Lima	4	+	+	-	-	+
FD01872698	1.207-2017	VNAU00000000	2017	Clinical	Lima	4	-	+	-	-	+

NA: Not available
+: Presence of the genes
-: Absence of the genes
